# Intraneural pseudocyst (so-called ganglion) in an unusual retroperitoneal periadnexal location?

**DOI:** 10.1186/1746-1596-9-150

**Published:** 2014-07-20

**Authors:** Dariusz Adamek, Carina Jägers, Maria Hejnold, Robert Jach, Bartlomiej Galarowicz

**Affiliations:** 1 Department of Pathomorphology, Jagiellonian University Medical College, ul. Grzegórzecka 16, Krakow 31-531, Poland; 2 Department of Gynecology and Oncology, Jagiellonian University Medical College, ul. Kopernika 23, Krakow 31-501, Poland

**Keywords:** Pseudocyst, Adnexa, Immunohistochemistry, Intraneural ganglion cyst, Ganglion cyst involving peripheral nerves (GCPN)

## Abstract

A case of an unusual unilocular cystic lesion of diameter 7 cm located retroperitoneally in the pelvis in close connection to the right adnexa of a 61 year-old woman is presented. Macroscopically, the lesion had a smooth outer and inner surface and was filled with translucent fluid. Histological examination revealed a fibrous and hyalinized wall which lacked a specific lining. Numerous nerve bundles in the cyst wall constituted the most conspicuous element of its histology possibly with some contribution of perineurial and/or mesothelial components. The morphology and immunohistochemistry speak for an intraneural pseudocyst sometimes called intraneural ganglion cyst which is rare in this location.

**Virtual Slides:**

The virtual slide(s) for this article can be found here: http://www.diagnosticpathology.diagnomx.eu/vs/1357862917132314

## Findings

Intraneural ganglion cysts or alternatively ganglion cysts involving peripheral nerves - GCPNs - (another synonym is intraneural pseudocyst) are rare disorders and present as unilocular cysts filled with translucent mucin [1]. Clinical features of these lesions typically include motor weakness, sensory changes or pain due to compression of the affected nerve [2]. Intraneural ganglion cysts affect most frequently the peroneal nerve but the involvement of other nerves of the upper and lower extremities as well as the obturator nerve have also been reported [2,3]. There had been lots of controversies over their pathogenesis until Spinner et al. suggested in 2003 in the now well accepted “unifying theory”, that an articular connection with the cyst and synovial fluid pushing its way through the nerve following the way of least resistance is the basic mechanism of cyst formation [4]. For every so far reported case of an intraneural ganglion cyst, the articular connection theoretically could be identified [5]. Here, an intraneural ganglion cyst located in the retroperitoneum in the vicinity of the right adnexa with no identifiable connection to a joint is reported. The presentation is enriched with a broadened discussion on the possible origins, differential diagnosis, and on histological contributors to the structure of its wall.

### Patient presentation

**Figure 1:**
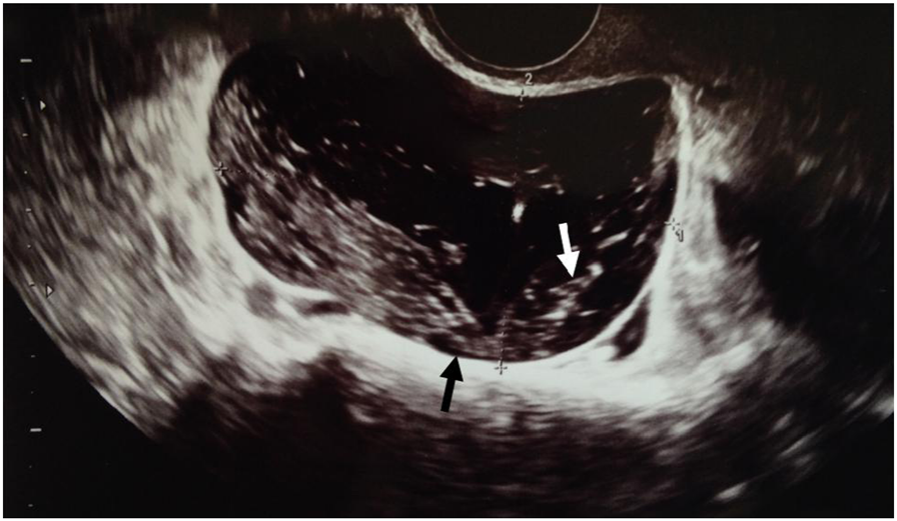
**Transvaginal ultrasonography.** The right adnexal region revealed a unilocular cyst 7 cm in maximal diameter with a thin wall (black arrow) and a mixed internal echogenicity (white arrow).

In the patient (woman, aged 61) with the history of ill-defined discomfort over the right lower abdomen and pelvis, the cystic lesion in the region of the right adnexa has been found in bimanual examination and in ultrasonography. She had no history of either previous operations, cesarean section or any other ailments or diseases. She had regular moderate menses (menarche at 14, menopause at 55) without complaints and bore 2 children by spontaneous delivery. The transvaginal ultrasonography at the admission to the Clinic of Gynecology and Oncology of the University Hospital in Krakow (Poland) did not reveal any other abnormalities or pathology in the pelvic region (uterus anteroflexed and normoechogenic) apart from the unilocular and oval, lemon-like shaped cyst of diameter 7 cm. The lesion presented with mixed echogenicity and without any abnormal vascularization in Power Doppler (PD) scan (Figure 1). The cyst visualized in laparoscopy was located in the retroperitoneum with connection to the external iliac vessels mimicking adhesion to the right adnexa. Noteworthy is the direct distance of the cyst wall to the hip joint which was less than 1 cm. It had a smooth surface and was filled with serous fluid. The cyst was enucleated and removed in Endobag™ with preservation of oncological sterility. The operation and postoperational follow-up were without complications. The patient was released home the day after procedure.

### Pathological examination

**Figure 2:**
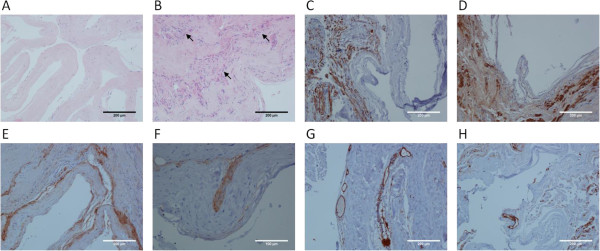
**Microscopic picture including immunohistochemical findings of the lesion. A**: The convoluted thin part of the cyst wall formed by hyalinized paucicellular connective tissue. Noteworthy is the lack of any distinct lining of the cyst. Hematoxylin Eosin staining. Objective magnification: 10×. **B**: Numerous nerve bundles present in the cyst wall (arrows). Hematoxylin Eosin staining. Objective magnification: 10×. **C**: Nerve bundles marked with antibody against PGP 9.5. Noteworthy is the arrangement of the nerve elements and the cyst lining that suggests an intraneural formation of the cyst. PGP 9.5 immunohistostaining. Objective magnification: 10×. **D**: The nerve bundles as if “embracing” the cyst wall. S-100 immunohistostaining. Objective magnification: 10×. **E**: EMA immunopositivity is present within the cyst wall but the very inner surface is totally negative. EMA immunohistostaining. Objective magnification: 10×. **F**: The pattern of immunopositivity for D2-40 strongly follows that of EMA in some regions, which suggests a co-localization. D2-40 immunohistostaining. Objective magnification: 20×. **G**: CD31 immunopositivity (similarly like CD34 – next figure) is limited to the endothelial elements. CD31 immunohistostaining. Objective magnification: 10×. **H**: Antibody against CD34 (like for CD31) marks only vascular endothelium. The lining of the cyst is negative. CD34 immunohistostaining. Objective magnification: 10×.

Macroscopically, the cyst had a smooth lining and the thickness of its wall varied from approximately 1 to 4 mm. Histologically, the wall of the cyst was formed by paucicellular connective fibrous and hyalinized tissue (Figure 2A) with a conspicuous admixture of nerve bundles (Figure 2B). In the slides immunostained for PGP 9.5 (Figure 2C) and S-100 (Figure 2D), the presence of nerve fibers in the majority of cross-sections through the cyst wall (see Figure 2D) was especially striking. The cyst did not show any form of conspicuous lining, neither of epithelial nor endothelial character (CD31 (Figure 2G) and CD34 (Figure 2H)). Numerous regions were strongly immunopositive for EMA (Figure 2E) and D2-40 (Figure 2F) within the cyst wall, but nowhere in the topmost inner surface of the cyst. The pattern of immunopositivity for EMA and D2-40 suggested even a mutual co-localization (compare Figure 2E and F). There were neither siderophages nor any hemosiderin deposits.

### Differential diagnosis

During the process of arriving at the diagnosis, several options arose and were evaluated (summarized in Table 1). Diagnosis *per exclusionem* is probably the optimal approach to such a (pseudo) cystic lesion like in our case.

**Table 1:** Differential diagnoses considered during the process of arriving at the diagnosis listed with their main histological characteristics, typical location, and basic references

**Differential diagnosis**	**Main histological characteristics**	**Location**	**References**
Intraneural ganglion cyst	Fibrous-walled, distorted nerve fascicles in the wall, non-epithelial lining	Proximity to joints in upper and lower extremities, common peroneal nerve	[1]
Posttraumatic neuroma	Haphazard bundles of regenerated axons, unencapsulated, nerve fibers within fibrous tissue	Stump of a transsected nerve, along the course of a traumatized nerve	[1]
Lymphangioma	Multiple variably sized cystic spaces, lymphoid endothelial lining, fibrocollagenous stroma	Various, including the ovary	[6]
Perineurial/Tarlov cyst	Peripheral nerves, frequently with arachnoidal membrane component	Nerve roots of the lower spinal cord	[7]
Mesothelial cyst	Lining of cuboidal or flattened mesothelial cells, fibrovascular stroma	Peritoneal Cavity	[8]
Endometriotic cyst	Fibrotic cyst wall, typically with remnants of endometrium and hemosiderin deposits, but occasionally without neither endometrial epithelium nor stroma (what justifies the term “hemorrhagic cyst of undetermined origin”)	Any site in abdomen and pelvis	[9]
other cystic lesions like: Pseudocystic metastasis of neuroendocrine tumor	Thick wall resembling the primary tumor, floating neoplastic cells, a blood-filled core, thin fibrous septa	Most frequently in the liver and regional lymph nodes	[10]

Regarding evident numerous nerve bundles in the wall of the cyst, the diagnosis of an intraneural ganglion cyst was initially favored. Intraneural ganglion cysts are unilocular cysts usually filled with translucent mucin and cause motor weakness and sensory changes or pain due to compression of the affected nerve [1,2]. Although intraneural ganglion cysts usually arise in the vicinity of joints, cysts in the pelvic region involving the obturator nerve with a connection to the hip joint have been reported [3,11]. In the presented case, a relation to the obturator nerve might explain the pathogenesis of the lesion in accordance with the articular theory [4]. Although not in the direct vicinity of the hip joint, intraneural ganglion cysts in the pelvic region can affect nerves which give rise to articular branches, including the sciatic, quadratus femoris, superior gluteal, obturator and femoral nerves [3]. However, a relation of the cyst either to the genital or to the femoral branch of the genitofemoral nerve might be more probable in the presented case. Although we don’t think that the cyst was related to the obturator nerve, one cannot exclude some atypical anatomical distribution of the nerves in the investigated case.

Haphazard and tortuous nerve bundles (Figure 2C) theoretically may also suggest associations with a form of a posttraumatic neuroma (in this case one may speculate of a “pseudocystic” form of it, Table 1).

Positivity for EMA – a reliable marker of perineurium [12] – may speak in favor of a perineurial cyst, which is frequently named Tarlov cyst (Table 1). Tarlov cysts typically occur in relation to intervertebral joints. However, they are reported in other locations including the pelvic region, where they mimic adnexal masses [13]. On the other hand, positivity for D2-40 arises the possibility that the cyst could be related to lymphatics [14]. Cystic forms of lymphangiomas (Table 1) were reported in the pelvic region [6]. When involving the ovary, lymphangiomas present as adnexal masses consisting of solid and cystic areas and can cause lower abdominal pain [6]. However, the positive immunostaining for both EMA and D2-40 in a similar expression pattern (Figure 2E and F) may in turn suggest a mesothelial character of the lesion [15] (mesothelial cyst, Table 1). Mesothelial cysts were also reported in this region [8]. Nevertheless, EMA and D2-40 positivity was not present in the very inner surface of the lesion but deeper within its wall.

Alternatively, one has to remember that there is a small chance for another extremely rare cystic form of a tumor, for example pseudocystic metastases of non-functioning gastro-entero-pancreatic neuroendocrine tumors (NETs) as reported by Fiori et al. [10]. Noteworthy is that also cystic metastases to the ovary with clinically overt primary tumors are common [16].

One should also not forget the possibility of a form of endometriotic cysts which not infrequently are devoid of any remnants of endometrium. However, typically they show evidences of previous hemorrhages (deposits of hemosiderin and/or siderophages). Such cysts deserve the term “hemorrhagic cysts of undetermined origin” [9]. In the presented case, not a single siderophage, nor any trace of hemosiderin that could suggest an endometriotic origin of the cyst were found.

Last but not least, one may speculate upon an intraneural, un-specific inflammatory process of pathogenesis. Although we have not found any evidences of an active inflammatory reaction (like lymphocytes etc.) one cannot exclude that some parts of S-100 immunopositivity may be due to the presence of dendritic cells, which are involved in autoimmune inflammatory processes within the nerve (autoimmune neuritis for instance) [17]. We may also hypothesize that an autoimmune inflammatory process terminated long ago could have lead to a cyst formation by creating osmotic pressure within the nerve. The same osmotic factor is probably also involved in synovial fluid pathogenesis of an intraneural cyst (ganglion) where synovium permeating the nerve bundle not only causes expansion of the nerve by pure hydrodynamic pressure but also acts by forming an osmotic force.Considering all aforementioned facts in the presented case, we think that there is no convincing evidence to attribute the lesion neither to perineurial nor lymphatic nor mesothelial origin. A development of the cyst inside the nerve seems to be especially convincing (Figure 2C and D) which makes the term “intraneural ganglion cyst” most suitable. However, immunopositivity for EMA and D2-40 in the presented case may suggest that there might be a contribution of perineurial and/or mesothelial elements to the structure of such pseudocysts located adjacent to the pelvic wall.

Lastly, one cannot help remarking that though it is very hard to find a more benign and innocuous lesion in human pathology in whichever site of the body as the cyst described here, even such a “simple” lesion demands cautious differential diagnosis. Moreover, a clinician, especially a gynecologist, being aware of the existence of lesions like reported here may be much more convincing trying to dispel any worries of a patient if confronted with a similar case.

## Consent

Written informed consent was obtained from the patient for publication of this Case Report and any accompanying images. A copy of the written consent is available for review by the Editor-in-Chief of this journal.

## Abbreviations

GCPN: Ganglion cyst involving peripheral nerves; EMA: Epithelial membrane antigen; NET: Gastro-entero-pancreatic neuroendocrine tumor.

## Competing interests

The authors declare that they have no competing interests.

## Authors’ contributions

DA made the diagnosis and edited the manuscript. CJ prepared a draft of the manuscript and performed critical review of literature. MH contributed to the histopathological examination and methods. RJ supervised the treatment of the patient and contributed to the description of the case. BG performed the physical examination and transvaginal ultrasonography and contributed its description to the manuscript. All authors read and approved the final manuscript.

## Authors’ information

D.A. (MD, PhD) is a specialist in pathomorphology and neuropathology and now at the position of Chair of Pathomorphology and Head of the Department of Neuropathology, Medical College, Jagiellonian University, Krakow (Poland). He is also a lecturer of pathology in the Faculty of Medicine of the Jagiellonian University.

C.J. (B.Sc.) is a student of biomedicine (master) at the Julius-Maximilians-University of Wuerzburg (Germany) doing an internship at the Chair of Pathomorphology, Jagiellonian University.

M.H. (MD) is a postgraduate/intern in pathomorphology at the Chair of Pathomorphology, Jagiellonian University.

R.J. (MD, PhD) is a specialist in Gynecology and Obstetrics and Gynecology and Oncology, Jagiellonian University. He is also a lecturer in Gynecology at the Medical Faculty of the Jagiellonian University.

B.G. (MD) is a postgraduate/intern at Gynecology and a junior assistant.
